# Clinical Aspects of Stevens-Johnson Syndrome and Toxic Epidermal Necrolysis With Severe Ocular Complications in South Korea

**DOI:** 10.3389/fmed.2021.640360

**Published:** 2021-02-22

**Authors:** Mee Kum Kim, Kyung Chul Yoon, Sook Hyun Yoon, Kyoung Yul Seo

**Affiliations:** ^1^Department of Ophthalmology, Seoul National University College of Medicine, Seoul, South Korea; ^2^Laboratory of Ocular Regenerative Medicine and Immunology, Seoul Artificial Eye Center, Seoul National University Hospital Clinical Research Institute, Seoul, South Korea; ^3^Department of Ophthalmology, Chonnam National University Medical School and Hospital, Gwangju, South Korea; ^4^Department of Ophthalmology, Institute of Vision Research, Yonsei University College of Medicine, Seoul, South Korea

**Keywords:** HLA-A*0206, HLA-B*5801, HLA-Cw*0303, HLA-Cw*0304, South Korea, Stevens-Johnson syndrome

## Abstract

This review describes the current knowledge regarding genetic susceptibilities and treatment strategies for Stevens-Johnson syndrome (SJS) and toxic epidermal necrolysis (TEN), with ocular complications, in Korea. In a case-control study, the gene frequencies of both HLA-A*0206 (20.0%) and HLA-Cw*0304 (15.0%) increased but the gene frequency of HLA-Cw*0303 (1.3%) decreased with cold medicine (CM)-SJS/TEN with severe ocular complications (SOCs). In a case-series, positive genotyping of HLA-B*5801 was 80.0% in allopurinol-induced SJS/TEN without SOCs. In a genome-wide association study, HLA-A*0206 was substantially related to CM-SJS/TEN with SOCs. Both HLA-A*0206 and prostaglandin-E receptor 3 (PTGER3) single nucleotide polymorphism (SNP) rs1327464 exert a synergistic effect on SOCs in CM-SJS/TEN. In the acute stage, conventional procedures, amniotic membrane transplantation or suture-less amniotic contact lenses are applied. Applications of intravenous Immunoglobulin (IVIG) or mega-dose steroids are attempted in patients with high acute ocular and systemic involvement scores. In the chronic stage, keratolimbal transplantation and penetrating keratoplasty are the standard procedures. Either autologous nasal or oral mucosal grafts, or biomaterial-free cultured oral mucosal epithelial cell sheets are transplanted as alternative therapies. Deep anterior lamellar keratoplasty is attempted. Combined photodynamic therapy with intrastromal bevacizumab injection or intense pulse laser are used to resolve chronic ocular complication. Corneoscleral contact lenses are available for a visual rehabilitation. As a last resort, Seoul-type keratoprosthesis had been transplanted. There are unmet needs to standardize nationwide ocular grading system and to correct tarsal scarring using mucosal grafting. This review provides a perspective on the current practices to treat ocular complications in SJS/TEN.

## Introduction

Steven-Johnson syndrome (SJS) and its severe form, toxic epidermal necrolysis (TEN), are severe, inflammatory vesiculobullous reactions of the skin and mucous membranes. The mortality rate of SJS and TEN are estimated as 1–10%, and 30%, respectively. According to the Korean National Health Insurance Database, SJS and TEN are infrequent, yet they constantly occur throughout the year by showing 3.96 and 0.94 cases per million/year for SJS and TEN, respectively (1). The management of SJS/TEN imposes a considerable clinical and financial burden, which is comparable with that of other major health problems (1, 2). SJS/TEN may permanently damage the affected mucosa, inducing severe sequelae including the lungs, genitals and eye. During primary intervention, acute ocular involvement occurs in approximately 60–100% of SJS/TEN patients (3–5). In Korea, ocular complications are reported as the most common complication related to SJS/TEN (1). Patients with ocular complications spent a considerable amount of money even after their recovery (2).

It is well known that SJS/TEN can be induced by various infections or classes of pharmacological agents, such as antibiotics, anticonvulsants, nonsteroidal anti-inflammatory drugs (NSAIDS), or allopurinol (6). Among the culprit drugs, as reported in a nationwide study, anticonvulsants are most frequent, followed by allopurinol, amoxicillin/dorzolamide, and acetaminophen (6). Previous pharmacogenomic studies demonstrated that certain human leukocyte antigen (HLA) genotypes could induce T-cell activation in response to a specific drug (7, 8). In a nationwide study that enrolled 5,802 Korean patients, allele frequencies of HLA-A*0206, HLA-B*5801, HLA-Cw*0303, and HLA-Cw*0304 were reported to be 10.3, 7.0, 10.9, and 9.1%, respectively (9). Specific genetic risk factors play an important role in the development of SJS/TEN. Recently, a genome-wide association study (GWAS) with a single nucleotide polymorphism (SNP) microarray has been employed to detect an association between SNPs and SJS/TEN (10, 11).

This review describes the current knowledge on the clinical aspect and treatment strategies for SJS/TEN, with ocular complications, in Korea. We summarized the HLA genotypes and the associated drugs for Koreans responsible for severe ocular complications (SOCs) in the acute and chronic stage of SJS/TEN, and elaborated upon the treatment strategies.

## Overview of Causative Drugs and Genetic Predisposition in Korean Patients With SJS/TEN With SOCs

Regarding frequencies of the culprit medications related with SOCs in Korea, cold medicine is the highest frequency, but antibiotics, allopurinol, or anti-epileptic drugs are not related to SOCs (12).

### Allopurinol

Allopurinol, a xanthine oxidase inhibitor, has been widely used to manage hyperuricemia and gout. Several studies report a strong association of the HLA-B*5801 genotype and allopurinol-induced SJS/TEN among Koreans (7–13%) (13–15). A recent study revealed that HLA-B75, DR13 homozygosity, or DR- 14 increased the risk of allopurinol-induced SJS/TEN when combined with HLA-B*5801, especially in patients with impaired renal function (14, 16). Compared to other drugs, allopurinol-induced SJS/TEN was associated with longer and more severe systemic manifestations, resulting in a high mortality rate (15); however, allopurinol-induced SJS/TEN may not cause serious acute or chronic ocular surface complications (12, 17). In a case-series, HLA-B*5801 genotype was observed in 80.0% of allopurinol-induced SJS/TEN without SOCs (17).

### Anti-epileptic Drugs

In a nationwide registry-based study, the most common causative AEDs were carbamazepine, lamotrigine, and levetiracetam (18, 19). In the case of AEDs-induced SJS/TEN, aromatic AEDs (e.g., carbamazepine, lamotrigine) were greatly associated with a severe reaction than non-aromatic AEDs (e.g., valproic acids) (19). It may be caused that AEDs containing an aromatic ring can form an arene-oxide intermediate, resulting in a hypersensitivity reaction (19). HLA-B*1502, which is closely related to SJS/TEN, is very rare in Koreans (7, 20). Several HLA genes are weakly associated with AEDs-induced hypersensitivity syndrome in the Korean population, but not with SJS/TEN (7, 20). Ocular manifestations are relatively mild in AEDs-induced SJS/TEN (12).

### Cold Medicine

Cold medicine (CM), including NSAIDS and acetaminophen, is relatively safe; however, it can trigger SJS/TEN in patients with suspected viral infection mediated by T-cells and monocytes (12, 21). SJS/TEN with severe ocular complications (SOCs) are commonly associated with CM in the Korean population (10, 22–25). In GWAS, HLA-A*0206 was considerably related to CM-SJS/TEN with SOCs (22). In addition, both HLA-A*0206 and prostaglandin-E receptor 3 (PTGER3) single nucleotide polymorphism (SNP) rs1327464 exert synergistic effect in CM-SJS/TEN with SOCs (23). A recent multicenter case-control study suggested that HLA-Cw*0304 may also be a positive marker for CM-SJS/TEN with SOCs; however, HLA-Cw*0303 may be an indicator of protection against this disease in the Korean population (24). In a worldwide GWAS that enrolled Korean, Japanese, Indian, and Brazilian patients, *IKAROS family zinc-finger 1* (IKZF1) was revealed as a novel susceptibility gene (meta-analysis, rs4917047) for CM-SJS/TEN with severe mucosal involvement (25).

### Carbonic Anhydrase Inhibitors

Surprisingly, both topical and oral formulations of CAIs, such as acetazolamide, methazolamide, and dorzolamide, can induce SJS/TEN (6, 26). HLA-B*5901 genotype, which occurs in 2.1% of the Korean population, has been suggested as a genetic marker for CAIs-induced SJS/TEN (27, 28). CAIs-induced SJS/TEN results in more extensive cutaneous manifestations and frequent ocular sequelae when compared with SJS/TEN due to other drugs including allopurinol, anticonvulsants, or anti-tuberculosis drugs (29).

### Other Drugs

Antibiotics such as amoxicillin/clavulanate and cephalosporin are the most common causative drugs in pediatric patients with SJS/TEN (30). However, there is no report stating that antibiotics may be related to SOCs (12). The anti-human immunodeficiency virus agents including abacavir and nevirapine could induce a hypersensitive reaction associated with HLA-B*5701 (7). However, HLA-B*5701 is not a clinically critical allele, since it is rare in Koreans (31).

## Treatment Strategy in Korean Patients With SJS/TEN With SOCs in the Acute Stage

General supportive care with anti-inflammatory intervention is the mainstay to restore barrier function of the skin and mucous membrane, and fluid balance, and to treat the infection (3, 10). The Korean severe cutaneous adverse drug reactions (SCARs) registry includes patients who were diagnosed with SJS and TEN (18, 32). Ocular involvement was in the ranges of 34–43% in Korea (1, 30). Therefore, therapeutic approaches to SJS/TEN should be multidisciplinary (3, 10). In Korea, patients with SJS/TEN are usually referred to the ophthalmologists upon presenting with complaints of ocular symptoms during hospitalization. Given that there is window of time within which vision-saving treatments can be applied, ophthalmologic consultation upon admission or within 24–48 h after diagnosis is critical (3, 10). The concept of a multidisciplinary approach, including eye care, should be shared with a primary physician.

There are local and systemic interventions to treat ocular complications in the acute stage of SJS/TEN (3, 10, 33, 34). As local treatments, aggressive lubrication, mechanical membrane removal/synechiolysis, bandage contact lens (CL) placement, and topical antibiotics and steroid application are implemented (3). Preservative-free artificial tears are instilled every 1–2 h and eyedrops containing hyaluronate are preferred in epithelial defected ocular surface. All membranes should be mechanically removed (33). However, there is no consensus about how often either membrane removal or synechiolysis be conducted since cotton-tip application can induce mechanical trauma. The benefits of mechanical synechiolysis should be cautiously weighed against the intervention-induced inflammation. One percentage topical prednisolone acetate combined with antiseptic eyedrops containing fluoroquinolone are preferably applied. 0.5–1.5% levofloxacin or 0.5% moxifloxacin eyedrops are administered three to four times a day. Topical 1% prednisolone acetate is administered every 2–3 h depending on the severity. High-oxygen-transmissible silicone hydrogel CL with medium water content (35–46%) such as Acuvue Oasys, Acuvue Advance, and PureVision are available to cover corneal epithelial defects (35).

Although there is no worldwide consensus on a grading system to assess severity of acute ocular involvement in SJS/TEN, new grading systems are currently being proposed (34, 36, 37). Sotozono et al. proposed a grading scale of 0–3 using three parameters, including conjunctival hyperemia, ocular surface epithelial defect, and pseudomembrane (36). Gregory et al. proposed four grading scales based on the presence of epithelial defected area with three parameters, including conjunctiva, cornea, and lid margin (37). In French, they reached a nationwide consensus on a diagnostic grading system for acute ocular complications that consists of three stages of severity using seven parameters (34). In Korea, we have not reached a consensus yet on grading system for acute ocular complications. The authors working with the international collaboration network of ocular SJS/TEN led by Kinoshita currently use the grading scales proposed by Sotozono (17, 38). Therefore, a nationwide consensus on the grading system to evaluate acute ocular complications should be established.

**Figure 1:**
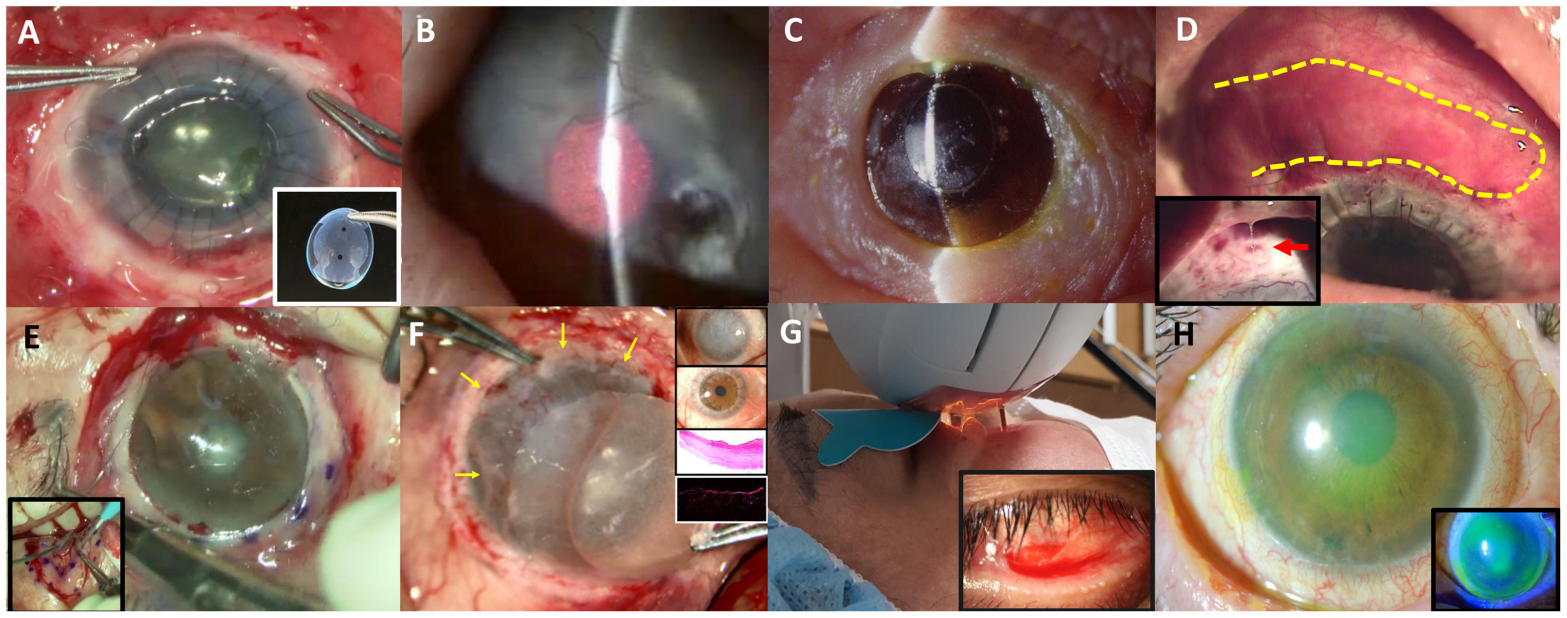
Various non-surgical and surgical modalities to treat ocular complications in Stevens-Johnson syndrome or Toxic epidermal necrosis are introduced. **(A)** Commercially available suture-less amniotic contact lens. **(B)** Photodynamic therapy with verteporfin combined with intrastromal bevacizumab injection. **(C)** Seoul-type keratoprosthesis. **(D)** Autologous nasal mucosal grafting (yellow-highlighted) combined with penetrating keratoplasty and keratolimbal allografting. A thick mucus is often secreted by the nasal mucosal graft (red arrow).* **(E)** Circumferential autologous oral labial mucosal grafting. **(F)** Biomaterial-free cultured oral mucosal epithelial cell sheets (COMECs) transplantation (yellow arrows) with H&E and K13 immunofluorescent staining. **(G)** Intense pulsed light. **(H)** Corneoscleral contact lens.

Amniotic membrane transplantation (AMT) is a standardized procedure for the severe acute ocular complication. AMT within the first 7–10 days can potentially avoid vision-threatening chronic complications (33, 37, 39). The indication of AMT includes (1) any corneal epithelial defect, (2) staining of the eyelid margin > 1/3 of its length, or (3) any conjunctival staining >1 cm at its greatest diameter and/or (4) pseudomembrane formation (33, 37, 39). There are two studies reporting the effect of AMT on visual improvement or SOCs in SJS/TEN in Korea (38, 40). One of the reports had presented the beneficial effect of AMT on visual improvement and SOCs (40). On the contrary, the other report showed that AMT was related with a poor final visual outcome; however, it did not mention when the AM was transplanted (38). AMT in the latter study may not be a timely treatment. The AMT technique to cover the whole ocular surface including fornix and the eyelid was recently standardized using the symblepharon ring and lid bolsters (39, 41, 42). In Korea, a similar technique of AMT was adapted. For a bedside application, ProKera is available in western countries (43), whereas suture-less amniotic membrane patch with a silicone ring (44) or suture-less amniotic CL is available in Korea. Effect of amniotic CL on wound healing was comparable to that of AMT *in vivo* study (Figure 1A, Supplementary Video 1) (45). However, the size of the amniotic CL is just enough to cover the cornea.

Systemic anti-inflammatory treatment is of utmost importance in reducing inflammation for both the body and the eye. So far, the effect of systemic intravenous immunoglobulin (IVIG) or mega-dose steroids on SOCs has been the subject of debate (38). In Korea, IVIG or mega-dose steroids are sometimes used for treatment of patients with high acute ocular and systemic involvement (38). A recent SCAR registry-based study showed that most of the patients have been treated with systemic steroid with an average maximal dose of 60 mg/day (18). Additionally, 87.5, 0.6, and 11.8% of the patients were treated using systemic steroids, IVIG, and both systemic steroids and IVIG, respectively (18); whereas, 49, 17, and 28% of the pediatric patients received systemic steroids, IVIG, and both systemic steroids and IVIG, respectively (30). It revealed that pediatric patients were more treated with IVIG compared to adults (18, 30). Considering that children with SJS/TEN have higher ocular and systemic complications than adults do (46, 47), such a treatment pattern may be reasonable. Combined treatment of IVIG and systemic steroids may be beneficial to reduce both SOCs and systemic complications.

## Treatment Strategy in Korean Patients With SJS/TEN With SOCs in the Chronic Stage

Complex ocular sequelae, such as symblepharon, lid malformation, trichiasis, conjunctival keratinization, limbal stem cell deficiency, corneal pannus, and dry eye occur in the chronic stage in patients with SJS/TEN. The goal of treatment in the chronic stage is the preservation of visual function, and reduction of the inflammation and persistent discomfort. Herein, surgical and medical interventions that have been currently practiced in Korea are presented (Table 1 and Figure 1).

**Table 1:** The clinical outcomes of various surgical interventions to treat chronic limbal stem cell deficiency in Korean SJS/TEN patients.

**First author (reference)**	**Enrolled patients (*N*)**	**Indications**	**Surgery**	Success^*^≥ 6 Ms (%)	**Visual improvement^**†**^ (%)**	**Mean FU (Ms) after surgery**
Han E. S. (48)	6	LSCD (± symblepharon)	KLAL (±PKP/AMT)	33.3	66.7	49.5
Choi S. E. (47)	3^‡^	LSCD	KLAL + PKP	33.3	NA	NA
Wee S. W. (49)	2	Failed previous PKP + corneal ulcer/opacification^**^	DALK + AMT (±additional PKP)	100	100	15
Kim M. K. (50)	6	Total LSCD + corneal opacity/symblepharon	S-KPro implantation	100	100^††^	46.8^ζ^
Chun Y. S. (51)	1	Total LSCD + corneal opacity/symblepharon	Autologous nasal mucosal grafting + PKP/KLAL	100	100	20
Choi H. R. (52)	4	Total or partial LSCD + corneal opacity (±symblepharon)	Autologous oral labial mucosal grafting (±PKP)	75	100	11
Kim Y. J. (53)	6	Total LSCD + corneal opacity	COMECs transplantation (±PKP)	83.3	66.7	8.8

*AMT, Amniotic membrane transplantation; COMECs, Biomaterial-free cultured oral mucosal epithelial cell sheets; DALK, Deep anterior lamellar keratoplasty; FU, follow-up; KLAL, Keratolimbal allograft; LSCD, Limbal stem cell deficiency; Ms, Months; N, Number; NA, not available data; PKP, penetrating keratoplasty; SJS, Stevens-Johnson syndrome; S-KPro, Seoul-type keratoprosthesis; TEN, Toxic epidermal necrolysis*.

* *Success; Success has been defined as the cornea has been well maintained without persistent epithelial defect at least 6 months after the surgery*.

† *Visual improvement has been defined as at least one-line increase of post-operative best corrected visual acuity during the follow-up compared with pre-operative best corrected visual acuity*.

‡ *Children SJS/TEN*.

** *This study did not mention whether LSCD was present or not*.

†† *The visual improvement has been assessed only in 3 SJS patients after exclusion of the other three patients who had a previous amblyopia, glaucoma, or retinal detachment*.

ζ *Primary retention time of S-KPro*.

### Medical Intervention Strategies

Chronic inflammation has been controlled by various medical interventions, such as serum eyedrops, topical or systemic steroid, and immunosuppressants. Autologous serum eye drops contain many anti-inflammatory molecules and are effective in reducing inflammation on the ocular surface (54); however, the components of serum eye drops may differ depending on the general condition of the patient. Steroid is effective but has undesirable adverse effects including glaucoma, cataract, infection, and delayed wound healing (55). Topical 0.02% tacrolimus ointment, originally approved for dermatologic purpose, is a good alternative to topical steroid (56). Topical 0.02% tacrolimus ointment was added to treat refractory chronic conjunctival inflammation in six SJS patients with tapering of the topical steroid (56). Topical tacrolimus decreased surface inflammation, corneal neovascularization, and intraocular pressure within 4 weeks (56).

Additionally, infection of the ocular surface should be closely monitored especially in SJS/TEN with SOCs. Unlike healthy people who show a high diversity of ocular microbiomes with prevalent streptococcus and lactobacillus, staphylococcus is a predominant bacteria with a less diversity with SJS, which can become easily pathogenic (57). A report in Korea presented higher rate of infective keratitis (35%) in LSCD with SJS than in those with a chemical burn (18%) (58). The higher the score of chronic ocular complications is, the more frequently infective keratitis develops in SJS (58).

Besides, tear film is unstable and meibomian gland is dysfunctional by the sequelae of SJS, and the degrees of meibomian gland disease tend to be correlated with the severity of SJS (59). Intense pulsed light is used to improve meibum expressibility (Figure 1G) (60). It contributes to decreased inflammatory cytokines such as IL-4,−6,−10, 17A, and TNF-α. IPL can be applied to stabilize tear film and reduce inflammatory cytokines, thereby treating severe meibomian gland obstruction in SJS.

Finally, corneoscleral CL with a total diameter of 14.0 mm is available for non-surgical visual rehabilitation in Korea (61). Fitting of a corneoscleral CL improved the vision by reducing corneal punctate erosions and reconstructing a new optical surface in six of eight SJS patients (Figure 1H) (61).

### The Outcome of Surgical Interventions

Intense immunologic reactions destruct limbal stem cells. Subsequently, corneal pannus occurs due to the loss of the limbal barrier function. Despite a high risk of rejection, keratolimbal allograft (KLAL) and penetrating keratoplasty (PKP) are standard procedures for visual rehabilitation. The clinical outcomes of various surgical interventions to treat chronic limbal stem cell deficiency (LSCD) in Korean SJS/TEN patients are shown in Table 1.

Eyes with SJS demonstrated a 33.3% of short-term success rate (≥6 months) and 16.7% of long-term success rate (≥2 years) in KLAL, which showed the least success rate among patients with LSCD (48). In children with SJS/TEN, LSCD developed in 32%, and combined PKP with KLAL failed in two (67%) out of three children (47). Deep anterior lamellar keratoplasty was attempted using acellular cornea with AMT in two eyes with previous failed PKP (49). One of them kept the cornea clear with epithelization. The other eye which needed additional PKP showed no additional corneal opacity (49). Photodynamic therapy with verteporfin combined with intrastromal bevacizumab injection was also applied to reduce corneal neovascularization (Figure 1B) (62). Within 6 months, five of eight eyes showed complete regression and the remaining eyes showed partial regression (62). In a few cases, Seoul-type keratoprosthesis (S-KPro) had been transplanted (Figure 1C) (50, 63). In the six S-KPro-implanted eyes of SJS, mean retention and visual preservation time was 46.8 and 35 months, respectively. To correct conjunctival keratinization with symblepharon or a LSCD, mucosal grafting has been attempted. A report presented successful reconstruction of the ocular surface and visual improvement by autologous nasal mucosal grafting accompanied with PKP and KLAL in a patient with SJS (Figure 1D) (51). Another report revealed visual improvement with a stable ocular surface by circumferential autologous oral labial mucosal grafting at the limbus in all four SJS patients (Figure 1E) (52). Recently, biomaterial-free cultured oral mucosal epithelial cell sheets (COMECs) transplantation has proven some efficacy on an LSCD in a clinical trial (Figure 1F) (53). Although the initial migration of the oral mucosal epithelial cells harvested from SJS patients (SJS-cells) was delayed with lower levels of epidermal growth factor and higher levels of vascular endothelial growth factor, compared to those of non-SJS cells, *in vivo* transplanted SJS-COMECs revealed similar expression of cytokeratin and stem cell markers as in non-SJS COMECs (64, 65). COMECs were transplanted in six SJS patients, and five eyes achieved complete reepithelization in a mean follow-up of 10.2 months (53). Among those five eyes, visual acuity was improved in four eyes with/without PKP (53).

Although the outcomes of various surgical interventions to treat LSCD cannot be directly compared due to different indications and follow-up periods (Table 1), autologous nasal or oral mucosal grafting, COMEC transplantation seem to show better successful outcome with visual improvement compared with that in KLAL. Meanwhile, first S-KPro implantation showed long-term successful outcome with visual improvement (50). However, due to skirt exposure, secondary exchange of S-KPro was mandatory in all S-KPro implanted patients (50). Given that retinal detachment developed in all S-KPro-exchanged eyes within 2 months (50), S-KPro implantation can be considered as a last resort. In Korea, less attention is paid to correction of scarring of the tarsal conjunctiva and lid malformation in SJS patients. There have been no reports regarding the reconstruction of tarsal scarring using a mucosal grafting in SJS yet.

## Discussion

In Korea, about 40% of the SJS/TEN patients suffer from chronic ocular complications. HLA-A*0206 combined with PTGER3 SNP rs1327464 enhances genetic susceptibility in CM-SJS/TEN with SOCs, whereas HLA-C*03:03 may be an indicator of protection against CM-SJS/TEN with SOCs. For the timely treatment of acute ocular complications, a nationwide consensus on ocular grading system should be reached, and a multidisciplinary approach including ophthalmologists should be standardized in Korea. In the chronic stage, various innovative surgical and medical modalities have been attempted to restore vision and stable ocular surface. Notably, both oral and nasal mucosal grafting as well as COMECs transplantation hold the most promise in the treatment of LSCD of Korean patients with SJS/TEN at present. However, the enrolled patient numbers were too small and the follow-up was too short to verify the long-term clinical efficacy. Therefore, large scale study with long-term follow-up should be further conducted. This review provides insightful information about genetic predisposition and current strategies to treat ocular complications of SJS/TEN in Korean population and gives us a perspective on how to improve current practice.

## Author Contributions

MK: conceptualization, data curation, formal analysis, investigation, methodology, resources, visualization, and roles/writing—original draft. KY: conceptualization, data curation, formal analysis, investigation, methodology, resources, and roles/writing—original draft. SY: data curation, formal analysis, investigation, visualization, and writing—editing. KS: conceptualization, formal analysis, supervision, validation, and writing—review and editing. All authors contributed to the article and approved the submitted version.

## Conflict of Interest

The authors declare that the research was conducted in the absence of any commercial or financial relationships that could be construed as a potential conflict of interest. The handling editor declared a past co-authorship with several of the authors MK, KY, and KS.
